# Flap Outcome Using “C” Shaped and “New” Incisions in Pediatric Cochlear Implantation

**Published:** 2012-04-01

**Authors:** M Ajalloueyan, S Amirsalari, Sh Radfar, A Tavallaee, S Khoshini

**Affiliations:** 1 Baqiyatallah Cochlear Implant Centre, Baqiyatallah University of Medical Sciences, Tehran, Iran

**Keywords:** Cochlear implant, Flap, Infection, Complication

## Abstract

**Background:**

Skin flap failure is a significant, though relatively uncommon complication of cochlear implant surgery. To achieve a good surgical result, a proper plan to locate the prospective implant is required. Thus, a new design of flap was evaluated in this regard.

**Methods:**

Two hundred and eleven consecutive children undergoing cochlear implantation in Baqiyatallah Cochlear Implant Center were compared with 75 cases who were operated through the classic “C shaped” fashion from Jul/14/2007 to Feb/14/2009.

**Results:**

There was one case of flap necrosis in the classic approach but there were no major flap complications in “new” design, also keloid formation as a minor complication was rare in the “new” method.

**Conclusion:**

The “new” design is easier to apply with fewer complications, so it can be recommended in children undergoing cochlear implantation.

## Introduction

**Fig. 1:**
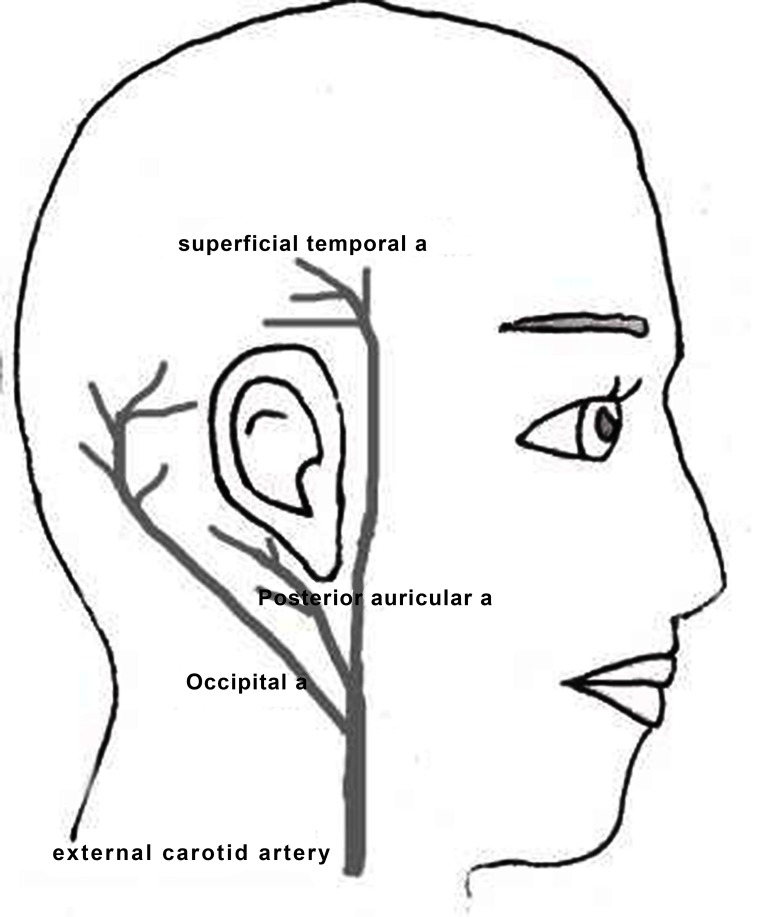
Vascular supply to the auricular area.

Prevention of scalp flap complication starts with a good flap design which should have adequate arterial supply, venous drainage, enough field exposure and coverage. To our knowledge, the blood supply to the field area involves mainly 3 branches off the external carotid artery including the post auricular, occipital and superficial temporal arteries (Figure 1). Venous drainage parallels with the arterial supply and gravity which facilitates this drainage. The incisions which impede arterial supply or gravitational venous drainage or cross the electrode and the receiver have the chance to break and fail short after the operation. Flaps that are too thick will impede electrical transmission and very thin flaps may erode under prosthesis or magnetic pressure, so flap thickness should be taken into consideration as an important item in children’s surgical planning. In kids who have a thin scalp, elevation of the periosteum along with the skin may protect the flap from the failure which may occur due to the implant pressure. Eliminating the large flaps avoids devascularization and minimizes the opportunity of flap infection or necrosis.

**Fig. 2:**
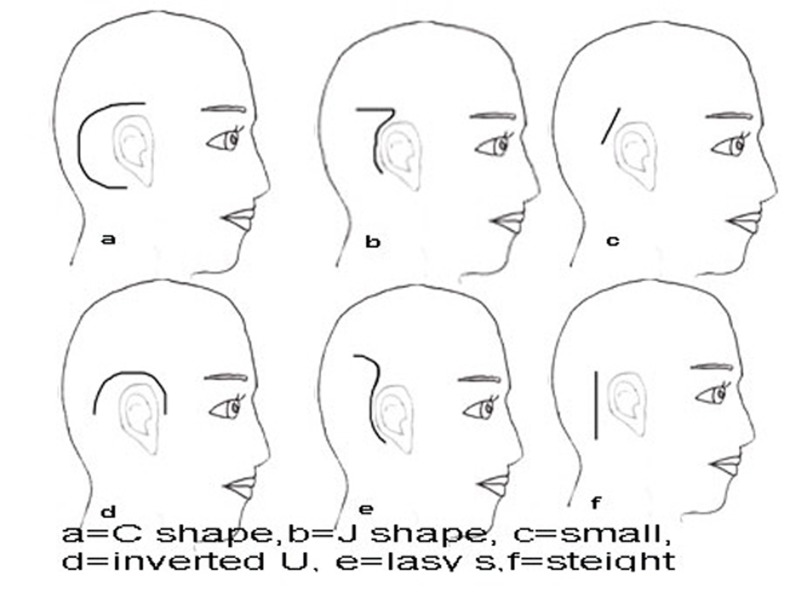
Conventional flap designs[9]

**Fig. 3:**
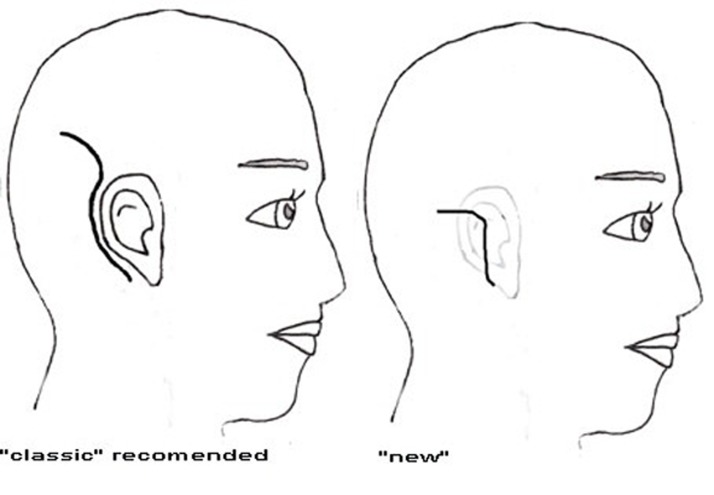
“C” shaped, ''new'' and “Hockey stick” flap designs[10]

According to the recent literature, the most frequently reported major cochlear implantation complications are related to the flap design. It also must be mentioned that the wound infection is reported in about 2-5% of the cases[1][2][3] undergoing conventional flaps. Most of the different incisions recommended for cochlear implantation were reviewed. The, ''J-shaped'', ''Straight''[4], ''lazy S''[5], ''endomeatal''[6], ''anteriorly based C-shaped'', ''inverted U-shaped'' and ''Hockey stick''[7] incisions were analyzed for their advantages of blood supply and cosmetic incision line[8] (Figure 2 and Figure 3).

## Materials and Methods

In Baqiyatallah Cochlear Implant Center from Jul/14/2007 to Feb/14/2009, 286 consecutive cases of children's cochlear implants were performed using Nucleus 24® and Nucleus freedom® (Cochlear Co.) by the author. The age range was limited to 1-6 years with 64% between 2-4 years and an equal distribution between male and female. In the first 75 patients, the classic “C”7 incision was used. After having a dreaded explantation due to flap necrosis in one case and the claims for knowing the cosmetics effects of the ''classic'' incisional line and occasional keloid formation, the ''classic'' flap was converted to the ''new'' above mentioned design. Baqiyatallah University Review Board for Medical Ethics approved the above mentioned work to be conducted in Baqiyatallah Hospital by the author in Jan/10/2008.

In ''new'' design, a cutaneus incision was carried out into the post auricular groove from the lowermost point of the pinna to the uppermost part of this sulcus. Then, according to the temporal line, it extends up-ward and backward to pass about two centimeters while keeping the periosteum intact (Figure 3). This incision prevents cutting off the trunk of the feeding vessels. Anteriorly based periosteal flap would be developed from one centimeter behind and underneath the skin incision to guaranty a good coverage of the receiver, the electrodes and the incision line. Through the above incision and proper exposure, simple mastoidectomy and a proper well for the receiver would be carried out through tangential drilling under the periosteum. After the introduction of the implant device, the accurate tensionless closure of the periosteum would be performed, using absorbable sutures. The skin was then closed in two separate layers and a light mastoidectomy dressing would keep the skin flap and consequently prevents formation of hematoma collection.

## Results

In the recent series of 211 cases, no major cutaneus infection or failure happened. All the patients`s family were satisfied about the cosmetic outcome of the new incision. Subcutaneous fluid collection or other minor complications did not occur. In the classic “C” shaped series, a child with ectodermal dysplasia developed severe reaction and flap necrosis, leading to rotation advancement of flap and skin graft in revision surgery and followed by explanation in the next intervention to save his life. Also, two cases developed subcutaneous serum or hematoma collection which responded to conservative therapy. All of 286 cases were treated with ceftrixone® (20 mg/kg, intra operatively) that continued for a day after the surgery.

## Discussion

All of the different incisions advocated for development of flaps, have advantages and disadvantages. The classic anteriorly based “ C-shaped” flap has the advantage of providing complete coverage of the device without crossing the implant, but this design has poor gravity based venous drainage and may lead to edema.[4] Inverted ''U-shaped'' and ''J-shaped'' flaps have the advantage of arterial supply and venous drainage but the incision line, crosses the electrode.[4] ''straight'', ''lazy S'' and ''endomeatal'' have limitations due to crossing the device,[9] and all have some limitations in exposure and application of the device.[8][11] The classic “C” incision takes the advantage of a good blood supply but has a large irregular and poor cosmetic incision line.[11] The “new” incision which is a modification of “Hockey stick” has smaller backward extension, so has less visible suture line and as anteriorly based periosteal flap guaranties secure coverage over the device, skin flap failure rate is guessed to be lower. The superiority of the ''new'' incision over the ''classic'' and others consists a complete intact blood supply and good soft tissue coverage over the device with less visible suture line.

While having less morbidity associated with the procedure, surgical complications such as scalp flap deficits, although very rare, but still occur, and executions to prevent the known mistakes may reduce the risk of flap complications by developing the flap designs. Table 1 compares the flap failures of different incisions. This ''new'' design is easy to perform while provides an appropriate vascular supply, a good field of exposure and coverage; without cosmetic problems.

**Table 1:** Sculp flap failure in pediatric cochlear implantation and different flap designs.

	**Author**	**No. of cases**	**Flap design**	**No. of failure**	**Percentage (%)**	**Note**
1	Ajalloueyan M	75	Classic^a^	1	1.3	Kids
2	Ajalloueyan M	221	''New''	0	0	Kids
3	Bahatia K[1]	300	?	7	2	Kids
4	Arnoldner C[2]	128	?	0	0	Kids
5	Sorrentino T[12]	487	Endaural	4	1	Kids and adults
6	Trinidade A[13]	371	?	5	1.3	Kids and adults
7	Telian SA[14]	116	Standard	6	10.3	Kids and adults
	Telian SA[14]	140	Post auricular	0	0	Kids and adults
8	Stratigouleas ED[9]	176	Straight^b^	0	0	Kids and adults
9	Calhoun D[5]	462	Lazy S	14	3	Kids and adults
10	O’Donoghue GM[15]	23	Straight	0	0	Kids
11	Gibson WP[4]	52	Straight	0	0	Kids
	Overall	2551		37	1.4	

^a^ classic is standard “C” shaped post auricular incision

^b^ bminimal incision
